# Assessment of organization of cervical and breast cancer screening programmes in the Latin American and the Caribbean states: The CanScreen5 framework

**DOI:** 10.1002/cam4.6492

**Published:** 2023-09-28

**Authors:** Isabel Mosquera, Clara B. Barajas, Li Zhang, Eric Lucas, Sara Benitez Majano, Mauricio Maza, Silvana Luciani, Partha Basu, Andre L. Carvalho

**Affiliations:** ^1^ Early Detection, Prevention & Infections Branch International Agency for Research on Cancer Lyon France; ^2^ Pan American Health Organization Washington DC USA; ^3^ Inequalities in Cancer Outcomes Network London School of Hygiene and Tropical Medicine London UK

**Keywords:** breast cancer, Caribbean, cervical cancer, Latin America, screening

## Abstract

**Background:**

In the Community of Latin American and Caribbean States (CELAC), breast cancer and cervical cancer are the first and third causes of cancer death among females. The objectives are to assess the characteristics of the cervical and breast cancer screening programmes in CELAC, their level of organization, and the association of screening organization and coverage of essential health services.

**Methods:**

Representatives of the Ministries of Health of 33 countries were invited to the CanScreen5 project. Twenty‐seven countries participated in a “Train The Trainers” programme on cancer screening, and 26 submitted data using standardized questionnaires. Data were discussed and validated.

The level of organization of the screening programmes was examined adapting the list of essential elements of organized screening programmes identified in a recently published IARC study.

**Results:**

Twenty‐one countries reported a screening programme for cervical cancer and 15 for breast cancer. For cervical cancer, 14 countries dedicated budget for screening (66.7%), and women had to pay in 3 countries for screening (14.3%), 9 for diagnosis (42.9%) and 8 for treatment (38.1%). Only 4 countries had a system to invite women individually (19.0%). For breast cancer, 8 countries dedicated budget for screening (53.3%), and women had to pay for screening in 3 countries (20.0%), diagnosis in 7 (46.7%) and treatment in 6 (40.0%). One country (6.7%) invited women individually.

There was variability in the level of organization of both cancer screening programmes. The level of organization of cervical cancer screening and coverage of essential health services were correlated.

**Conclusion:**

Large gaps were identified in the organization of cervical and breast cancer screening services. CELAC governments need pragmatic public health policies and strengthened health systems. They should guarantee sustainable funding, and universal access to cancer diagnosis and treatment. Moreover, countries should enhance their health information system and ensure adequate monitoring and evaluation.

## 1 INTRODUCTION

In the Community of Latin American and Caribbean States (CELAC), breast cancer and cervical cancer are the first and third most common causes of cancer death among females (13.5 and 7.6/100,000 person‐years, respectively).^1^ Mortality from either cancer can be decreased through effective implementation of a well‐organized population‐based screening of eligible women.^2^, ^3^

Earlier reports on the screening programmes for cervical and breast cancer in CELAC have focused mainly on their protocol (screening test, target age, and interval) using secondary sources, and on their examination coverage derived from population‐based surveys or published studies.^4^, ^5^, ^6^, ^7^ Performance reports of the screening programmes in the region are rarely published by the health authorities,^8^ and even when they do, they cover only information on screening in the public sector. A working group comprising of global experts in cancer screening convened by the International Agency for Research on Cancer (IARC/WHO) recently defined 16 essential elements for organized screening.^9^ Therefore, it is of interest to assess the characteristics of screening programmes in CELAC against the essential elements for programme organization.

Within the CanScreen5 project, screening programmes are considered those with a commitment from the government to provide the screening services to the eligible population as defined by laws, statutes, regulations, or official notifications, and with a screening protocol in place.^10^ To the best of our knowledge, there is a lack of detailed reports on the organization of cervical and breast cancer screening programmes using standardized tools to collect data and information directly from the programmes across countries in CELAC. Therefore, the objectives of this paper are to assess: (a) the characteristics of the cervical and breast cancer screening programmes in CELAC using a common methodology, (b) the level of organization of cervical and breast cancer screening programmes in CELAC, and (c) the association of screening organization and coverage of essential health services.

## 2 METHODS

IARC in collaboration with Pan‐American Health Organization (PAHO/WHO) approached the Ministries of Health (MoH) of 33 countries to identify and nominate experts responsible of cancer screening implementation to participate in the CanScreen5 (Cancer Screening in Five Continents) project. The CanScreen5 project aims to collect information on the characteristics and performance of cancer screening programmes across the globe in a standardized manner for an effective programme evaluation and quality improvement.^10^, ^11^, ^12^ Collaborators from 27 countries participated in a “Train The Trainers” (TTT) programme, designed to create a network of master‐trainers capable of training health care providers on cancer screening programme monitoring, evaluation and quality improvement. This programme was not a requirement to fill in questionnaires for the first 12 countries, as it was developed after their recruitment, but was for the other 15.

The TTT programme took place during 2020–2023, and included online self‐paced learning modules (1.5–2 h/each) in Spanish and English (publicly available as self‐paced training).^13^ The training programme covered the following topics: principles of cancer screening, planning and implementing a cancer screening programme, and assuring quality of such programmes. The blended model of TTT also included up to six biweekly 2‐h live sessions, group assignments and face‐to‐face workshops. These live sessions did not include training on how to train, this was approached in subsequent meetings. Each master–trainer has free access to all online resources so that she/he can train more participants.

Out of 27 countries participating in training, 26 (96.3%) submitted data using standardized questionnaires on qualitative information on their cervical and breast cancer screening programmes, including their organization, screening protocol, invitation and follow up mechanisms, and system of quality assurance. Countries submitting data were the following: El Salvador, Guatemala, Honduras, Mexico, Nicaragua, and Panama in Central America; Argentina, Brazil, Chile, Colombia, Ecuador, Guyana, Paraguay, Peru, Suriname, and Uruguay in South America; and Antigua and Barbuda, Bahamas, Cuba, Dominica, Dominican Republic, Grenada, Jamaica, Saint Kitts and Nevis, Saint Lucia, and Saint Vincent and the Grenadines (SVG) in the Caribbean. Countries were considered to have a screening programme in place if at least they had a documented policy recommending cancer screening and a documented screening protocol. Data were collected on the CanScreen5 data submission platform.

Countries were asked to provide quantitative data on the performance of the programme, which was only accepted if at least they could provide the number of women screened and screening test outcomes. Data collection covered the number of screened populations, screening test results, compliance to further assessment, final diagnosis, cancer staging, and treatment from the same cohort of screen eligible women, either for a year or a screening round. Quantitative data were collected and managed using REDCap (Research Electronic Data Capture) electronic data capture tools hosted at IARC. REDCap is a secure, web‐based software platform designed to support data capture for research studies, providing an intuitive interface for validated data capture, audit trails for tracking data manipulation and export procedures.^14^, ^15^

Qualitative data of 26 countries were reviewed by the IARC Secretariat (2–3 researchers), and cross‐checked against policies, cancer control plans, protocols and guidelines, documents on quality assurance, and published reports. After internal validation, data were submitted to the CanScreen5 Scientific Committee (SC) for verification by 2 members, with a third if needed. Once the SC validated the data, countries were informed and data were published on the CanScreen5 web portal.^10^ Regarding quantitative data, the current manuscript reports only whether there was a system of data collection to evaluate performance of the breast and cervical cancer screening programmes and whether the data was available for analysis.

Qualitative data were used to assess the level of organization of the screening programmes adapting the list of essential elements of organized screening programmes identified in the recently published IARC study.^9^ In that expert consensus, 16 essential elements were identified, and 13 of them were covered in the tools already in use in the CanScreen5 project. These include: (1) existence of a policy recommending screening, (2) having a system to identify eligible population, (3) having a system collecting data on screening test results and further assessment, (4) linkage of screening data with population‐based cancer registry (PBCR), (5) existence of an evidence‐based protocol/guideline that is universally complied with, (6) description of primary screening test, target age and screening interval, (7) mechanism to send individual invitations to eligible population, (8) mechanism to actively contact screen positive population, (9) existence of a policy on quality assurance of screening delivery, (10) person/team responsible for quality assurance, (11) specified performance indicators, (12) reference standards of these indicators, and (13) publication of performance reports in last 5 years. The criteria not evaluated in the tools were monitoring and evaluation being covered in the protocol, auditing of the programme, and provision for continued training for service providers. The level of organization for each cancer site and country for the reported year was calculated as a proportion of the 13 essential elements included in the tools that were fulfilled by each programme and categorized in quartiles.

Coverage of essential health services was measured with the universal health coverage (UHC) service coverage index. This index is defined as the average coverage of essential services based on indicators including reproductive, maternal, new‐born and child health; infectious diseases; noncommunicable diseases (NCD); and service capacity and access, among the general and most disadvantaged population, dimensions each with their corresponding subindex.^16^ Cervical cancer screening coverage is among the indicators considered to calculate the UHC service coverage index. We examined if there was a correlation between the level of organization of screening programmes and the UHC service coverage index. We calculated the *p* value using the Spearman correlation method with SPSS.

## 3 RESULTS

Out of the 26 countries, 21 had cervical cancer screening programmes, 6 being in Central America, 9 in South America, and 6 in the Caribbean. According to our definition, 15 countries had breast cancer screening programmes, covering 4 countries in Central America, 7 in South America, and 4 in the Caribbean. Antigua and Barbuda, Dominica and Saint Lucia had neither cervical nor breast cancer programme, and Bahamas, Guatemala, Honduras, Guyana, Grenada, and Saint Kitts and Nevis did not have a breast cancer screening programme, while Ecuador had pilots in screening for both cancer sites.

### 3.1 Policies, budget, and payment for services

Cervical cancer screening programmes began earlier than breast ones, Cuba being the pioneer in the region (1968 for cervix and 1990 for breast, Tables 1 and 2). The policy supporting the implementation of the cervical cancer programme was in the form of a law in Chile, Nicaragua, and Paraguay, while the rest of the countries had an official notification from the MoH/Health Authority, published in the government official publication (official journal, official gazette, official newspaper, official bulletin, etc.). Exceptions were Bahamas, Guatemala, Guyana, and Suriname, that only had a recommendation from a public institution/professional organization/association and endorsed by the MoH/Health Authority. There was a cervical cancer screening programme coordinator in most countries (*n* = 17; 80.1%), among them the 9 South American countries. MoH had a dedicated budget for cervical cancer screening in 14 countries (66.7%), and screening was free of charge in 18 countries (85.7%) and reimbursed in 1. Out of pocket expenditure was needed in 9 countries for diagnosis (42.9%) and in 8 for treatment (38.1%).

**TABLE 1:** Cervical cancer screening organization in CELAC by country.

Country (reporting year)	Year of progr. initiation	Documented screening policy	Dedicated budget	Screening free of charge	Diagnosis free of charge	Treatment free of charge
*Central America*						
El Salvador (2020)	‐	Notification	✓	✓	✓	✓
Guatemala (2021)	2013	Recommendation	x	✓	✓	✓
Honduras (2020)	1997	Notification	✓	✓	x	✓
Mexico (2021)	‐	Notification	✓	✓	✓	✓
Nicaragua (2019)	1983	Law	✓	✓	✓	✓
Panama (2020)	1980	Notification	x	✓	x	x
*South America*						
Argentina (2022)	2012	Notification	✓	✓	✓	✓
Brazil (2020)	1988	Notification	✓	✓	✓	✓
Chile (2020)	1986	Law	✓	✓	x	x
Colombia (2020)	2000	Notification	✓	✓	✓	✓
Guyana (2021)	2009	Recommendation	✓	✓	✓	✓
Paraguay (2020)	2007	Law	✓	✓	✓	✓
Peru (2020)	1990	Notification	✓	✓	✓	✓
Suriname (2021)	1975	Recommendation	x	x*	x	x
Uruguay (2021)	1995	Notification	x	✓	x	x
*The Caribbean*						
Bahamas (2020)	‐	Recommendation	✓	✓	x	x
Cuba (2021)	1968	Notification	✓	✓	✓	✓
Dominican Republic (2021)	2021	Notification	✓	x	x	x
Grenada (2021)	‐	Notification	x	✓	x	x
Jamaica (2021)	2004	Notification	x	✓	✓	✓
Saint Vincent and the Grenadines (2021)	‐	Notification	x	x	x	x

*Note*: Documented screening policy was defined as follows: law (signed by the president or approved by the parliament); notification from Ministry of Health/Health Authority (published in the government official publication ‐official journal, official gazette, official newspaper, official bulletin, etc.‐); or recommendation (from public institution/professional organization/association and endorsed by the Ministry of Health/Health Authority).

* Fully reimbursed by insurance.

**TABLE 2:** Breast cancer screening organization in CELAC by country.

Country (reporting year)	Year of progr. initiation	Documented screening policy	Dedicated budget	Screening free of charge	Diagnosis free of charge	Treatment free of charge
*Central America*						
El Salvador (2020)	‐	Notification	x	✓	✓	✓
Mexico (2021)	2001	Notification	✓	✓	✓	✓
Nicaragua (2019)	2006	Law	✓	✓	✓	✓
Panama (2020)	2008	Notification	x	✓	x	x
*South America*						
Argentina (2022)	‐	Notification	✓	✓	✓	✓
Brazil (2020)	2004	Notification	✓	✓	✓	✓
Chile (2020)	1995	Law	✓	✓	x	x
Colombia (2020)	2000	Notification	✓	✓	✓	✓
Paraguay (2020)	2007	Law	✓	✓	✓	✓
Suriname (2021)	‐	Recommendation	x	x*	x	x
Uruguay (2021)	‐	Notification	x	✓	x	x
*The Caribbean*						
Cuba (2021)	1990	Notification	✓	✓	✓	✓
Dominican Republic (2021)	2021	Notification	x	x	x	x
Jamaica (2021)	‐	Notification	x	x	x	✓
Saint Vincent and the Grenadines (2021)	‐	Notification	x	✓	x	x

*Note*: Documented screening policy was defined as follows: law (signed by the president or approved by the parliament); notification from Ministry of Health/Health Authority (published in the government official publication ‐official journal, official gazette, official newspaper, official bulletin, etc.‐); or recommendation (from public institution/professional organization/association and endorsed by the Ministry of Health/Health Authority).

* Fully reimbursed by insurance.

Chile, Nicaragua, and Paraguay had a law in place supporting implementation of breast cancer screening. The rest of the countries had a notification, except for Suriname having a recommendation. Compared to cervical cancer screening, a similar proportion of countries had a dedicated programme coordinator (*n* = 12; 80.0%). On the other hand, a lower proportion of countries had a dedicated budget to implement breast cancer screening (*n* = 8; 53.3%). Women had to pay for breast cancer screening in 3 countries (20.0%) and were reimbursed for the service in 1. Women had to pay for diagnosis in 7 (46.7%) and for treatment in 6 (40.0%) countries. Countries with both cervical and breast cancer programmes displayed the same pattern of out‐of‐pocket expenditure, that is, if screening, diagnosis and/or treatment had to be paid for in the cervical cancer screening programme, they also had to be paid for in the breast cancer screening programme. The only exceptions were Jamaica, where screening and diagnosis were for free for cervical cancer, but not for breast cancer, and SVG, with breast cancer screening being free of charge, but not for cervical cancer.

In Central America there was a dedicated budget in 66.7% of countries for cervical cancer and 50.0% for breast cancer, and screening was free of charge for both cancer sites. South America was the region with a higher proportion of countries with a dedicated budget for screening, and most of the countries screened women free of charge. In the Caribbean only half of the countries had a dedicated budget for cervical cancer screening and 25% for breast cancer, and in this region, women were more likely to have to pay for screening, diagnosis, and treatment of cervical and breast cancer.

### 3.2 Screening protocol

There was a great variability in the screening methods, interval, and target age (Table 3 and 4). For cervical cancer, only 8 countries (38.1%) used a single primary test. Availability of the test determined the choice of screening test used in countries having multiple tests included in their protocol. The most frequently reported screening method was cytology every 3 years (*n* = 13; 61.9%). Eight countries (38.1%) had HPV testing as a primary screening method, while co‐testing was available in Bahamas, Chile, and Panama (14.3%). Seven countries screened with VIA (33.3%). Triage methods used were cytology for positive HPV test in Argentina, Colombia, Mexico, and Paraguay; HPV to triage abnormal cytology in Dominican Republic, and Suriname; and VIA to triage HPV positive in El Salvador and Guatemala. El Salvador, Honduras, Nicaragua, and Suriname had no maximum target age for cervical cancer screening.

**TABLE 3:** Screening protocol for cervical cancer in CELAC by country.

Country (reporting year)	Primary screening test(s)	Target age (years)	Screening interval
*Central America*			
El Salvador (2020)	Cytology	≥20	2 years
	HPV	30–59	5 years
Guatemala (2021)	Cytology	25–54	3 years
	VIA	25–40	3 years
	HPV	30–49	5 years
Honduras (2020)	VIA	^a^−49	3 years
	Cytology	≥50	1 year
Mexico (2021)	Cytology	25–34	3 years
	HPV	35–64	5 years
Nicaragua (2019)	Cytology	≥15	1 year
Panama (2020)	Cytology	21–64	2 years
	HPV	25–64	3 years
	HPV & cytology (co‐test)	30–64	3 years
*South America*			
Argentina (2022)	Cytology	25–70	3 years
	HPV	30–64	5 years
Brazil (2020)	Cytology	25–64	3 years
Chile (2020)	Cytology	25–64	3 years
	HPV & cytology (co‐test)	30–64	3 years
Colombia (2020)	Cytology	25–29	3 years
	HPV	30–65	5 years
	VIA	30–50	3 years
Guyana (2021)	VIA	25–49	3 years
Paraguay (2020)	Cytology	^b^−65	1 year
	HPV	30–64	5 years
Peru (2020)	Cytology	25–64	2 years
	HPV	30–49	5 years
	VIA	30–49	2 years
Suriname (2021)	VIA	≥23	1 year
	Cytology	≥50	3 years
Uruguay (2021)	Cytology	21–69	3 years
*The Caribbean*			
Bahamas (2020)	Cytology	21–65	3 years
	Cytology	21–29	3 years
	HPV & cytology (co‐test)	30–65	5 years
Cuba (2021)	Cytology	25–64	3 years
Dominican Republic (2021)	Cytology	25–60	1 year
Grenada (2021)	Cytology	21–55	3 years
	VIA	21–55	3 years
Jamaica (2021)	Cytology	21–64	3 years
Saint Vincent and the Grenadines (2021)	Cytology	21–65	3 years

Abbreviations: HPV, human papillomavirus; VIA, visual inspection with acetic acid.

^a^ Sexual debut.

^b^ One year after sexual debut.

**TABLE 4:** Screening protocol for breast cancer in CELAC by country.

Country (reporting year)	Primary screening test(s)	Target age (years)	Screening interval
*Central America*			
El Salvador (2020)	Mammography	40–69	1 year
Mexico (2021)	CBE	25–39	1 year
	Mammography	40–69	2 years
Nicaragua (2019)	Mammography	40–49	2 years
		≥50	1 year
Panama (2020)	Mammography & US (co‐test)	40–74	2 years
*South America*			
Argentina (2022)	Mammography	50–69	2 years
Brazil (2020)	Mammography	50–69	2 years
Chile (2020)	Mammography	50–69	3 years
Colombia (2020)	CBE	40–49	1 year
	Mammography	50–69	2 years
Paraguay (2020)	Mammography	40–65	1 year
Suriname (2021)	Mammography	50–75	2 years
Uruguay (2021)	Mammography	50–69	2 years
*The Caribbean*			
Cuba (2021)	CBE	≥30	1 year
Dominican Republic (2021)	Mammography	40–65	1 year
Jamaica (2021)	Mammography	40–69	1 year
Saint Vincent and the Grenadines (2021)	Mammography	45–54	1 year
		≥55	2 years

Abbreviations: CBE, clinical breast examination, US, ultrasound.

Breast cancer screening was conducted with mammography every 2 years in 8 countries (53.3%). All countries kept the same screening interval across all eligible age groups, except Nicaragua and SVG, which had different intervals based on age. All eligible women in Cuba were screened with clinical breast examination (CBE), while Colombia and Mexico used this method in the younger women only. Women in Panama were screened with both mammography and ultrasound. Screening began at age 40 or 50 years, except in Mexico, Cuba and SVG, where the age at onset of screening was 25, 30, and 45 years, respectively. All countries set a maximum target age for breast cancer screening, except Cuba, Nicaragua and SVG.

### 3.3 Invitation, follow‐up and quality assurance

Only 4 countries (19.0%) had a system in place to invite women individually for cervical cancer screening (Table 5). The source to identify eligible women was a list from primary care or family physicians (Colombia, Cuba, El Salvador, and Nicaragua), population register (Nicaragua), and insurance company list (Colombia). In all 4 countries invitation was conducted through home visits by health workers, and in Colombia women were also invited through phone calls, SMS, and emails. Chile invited only women due to participate in screening on a given period who did not undergo screening. Eleven countries (52.4%) tracked screen‐positive women and 12 (57.1%) tracked precancer and cancer cases to ensure their compliance to further management. Half of these were countries in Central America.

**TABLE 5:** Cervical cancer screening invitation, follow up and quality assurance (QA) in CELAC by country.

Country (reporting year)	System of individual invitation	Tracking of screen positives	Tracking of women with precancers/cancers	Individual/team responsible for QA	Documented performance indicators	Evaluation reports published in last 5 years
*Central America*						
El Salvador (2020)	✓	✓	✓	✓	✓	x
Guatemala (2021)	x	✓	✓	x	✓	x
Honduras (2020)	x	✓	✓	x	✓	x
Mexico (2021)	x	✓	✓	✓	✓	✓
Nicaragua (2019)	✓	✓	✓	✓	✓	x
Panama (2020)	x	✓	✓	x	✓	x
*South America*						
Argentina (2022)	x	✓	✓	✓	✓	x
Brazil (2020)	x	x	x	x	✓	✓
Chile (2020)	x	✓	✓	✓	✓	✓
Colombia (2020)	✓	✓	✓	✓	✓	✓
Guyana (2021)	x	x	x	✓	✓	x
Paraguay (2020)	x	x	x	x	x	x
Peru (2020)	x	x	x	✓	✓	x
Suriname (2021)	x	x	x	x	x	x
Uruguay (2021)	x	x	x	✓	✓	✓
*The Caribbean*						
Bahamas (2020)	x	x	x	x	x	x
Cuba (2021)	✓	✓	✓	✓	✓	x
Dominican Republic (2021)	x	✓	✓	x	✓	x
Grenada (2021)	x	x	x	x	x	x
Jamaica (2021)	x	x	x	✓	✓	x
Saint Vincent and the Grenadines (2021)	x	x	✓	x	x	x

Eleven of the cervical cancer screening programmes (52.4%) had a person or a team responsible for quality assurance, which took into account public providers, and most had documented performance indicators (*n* = 16, 76.2%), including the 6 countries in Central America. Out of the 16 countries reporting the use of performance indicators, 3 (14.3%) did not have specified reference standards for the indicators. However, only 5 countries (23.8%) had published evaluation reports in the last 5 years, none of these countries were in the Caribbean. Fifteen countries had a PBCR (71.4%), but none had a linkage between the PBCR and the cervical cancer screening registry.

Regarding breast cancer screening, only one country (6.7%) had a system to invite women individually (Table 6). Cuba used the list of primary care or family physicians to identify eligible women and invited them through home visits by health workers. Chile, Colombia, and SVG invited only women due to participate in screening in a given period who had not undergone screening. Nine countries tracked screen‐positive women and cancer cases (60.0%).

**TABLE 6:** Breast cancer screening invitation, follow up and quality assurance (QA) in CELAC by country.

Country (reporting year)	System of individual invitation	Tracking of screen positives	Tracking of women with cancers	Individual/team responsible for QA	Documented performance indicators	Evaluation reports published in last 5 years
*Central America*						
El Salvador (2020)	x	✓	✓	✓	x	x
Mexico (2021)	x	✓	✓	✓	✓	✓
Nicaragua (2019)	x	✓	✓	✓	✓	x
Panama (2020)	x	✓	✓	x	x	x
*South America*						
Argentina (2022)	x	x	x	✓	✓	x
Brazil (2020)	x	x	x	✓	✓	✓
Chile (2020)	x	✓	✓	✓	✓	✓
Colombia (2020)	x	✓	✓	✓	✓	✓
Paraguay (2020)	x	x	x	x	x	x
Suriname (2021)	x	x	x	✓	x	x
Uruguay (2021)	x	‐	‐	x	x	x
*The Caribbean*						
Cuba (2021)	✓	✓	✓	✓	x	x
Dominican Republic (2021)	x	✓	✓	x	x	x
Jamaica (2021)	x	x	x	✓	x	x
Saint Vincent and the Grenadines (2021)	x	✓	✓	x	x	x

Compared to cervical cancer screening, a higher proportion of breast cancer screening programmes had a person or a team responsible for quality assurance (*n* = 10; 66.7%), but a lower proportion of countries (*n* = 6, 40.0%) had documented performance indicators. All these 6 countries had set reference standards for the indicators (40.0%), with 4 (26.7%) having published reports in the last 5 years. No country had their breast cancer screening registry linked to the PBCR, even though PBCR existed in 11 countries (73.3%).

In Central America screen positive and cancer cases were tracked for both cancer sites. All countries in this region had documented performance indicators for cervical cancer, and only half for breast cancer, with only 1 country publishing an evaluation report in the last 5 years for either cancer site. Women in South America were less likely to be tracked in case of a screen‐positive cervical or breast cancer case. Performance indicators were specified in 77.8% of the South American countries for cervical cancer and 57.1% for breast cancer, and 44.4% had published an evaluation report on cervical cancer and 42.9% on breast cancer in the last 5 years. In the Caribbean there was rarely someone responsible for quality assurance and no country had published an evaluation report in the last 5 years for either cervical or breast cancer.

### 3.4 Level of organization and UHC services coverage

**FIGURE 1:**
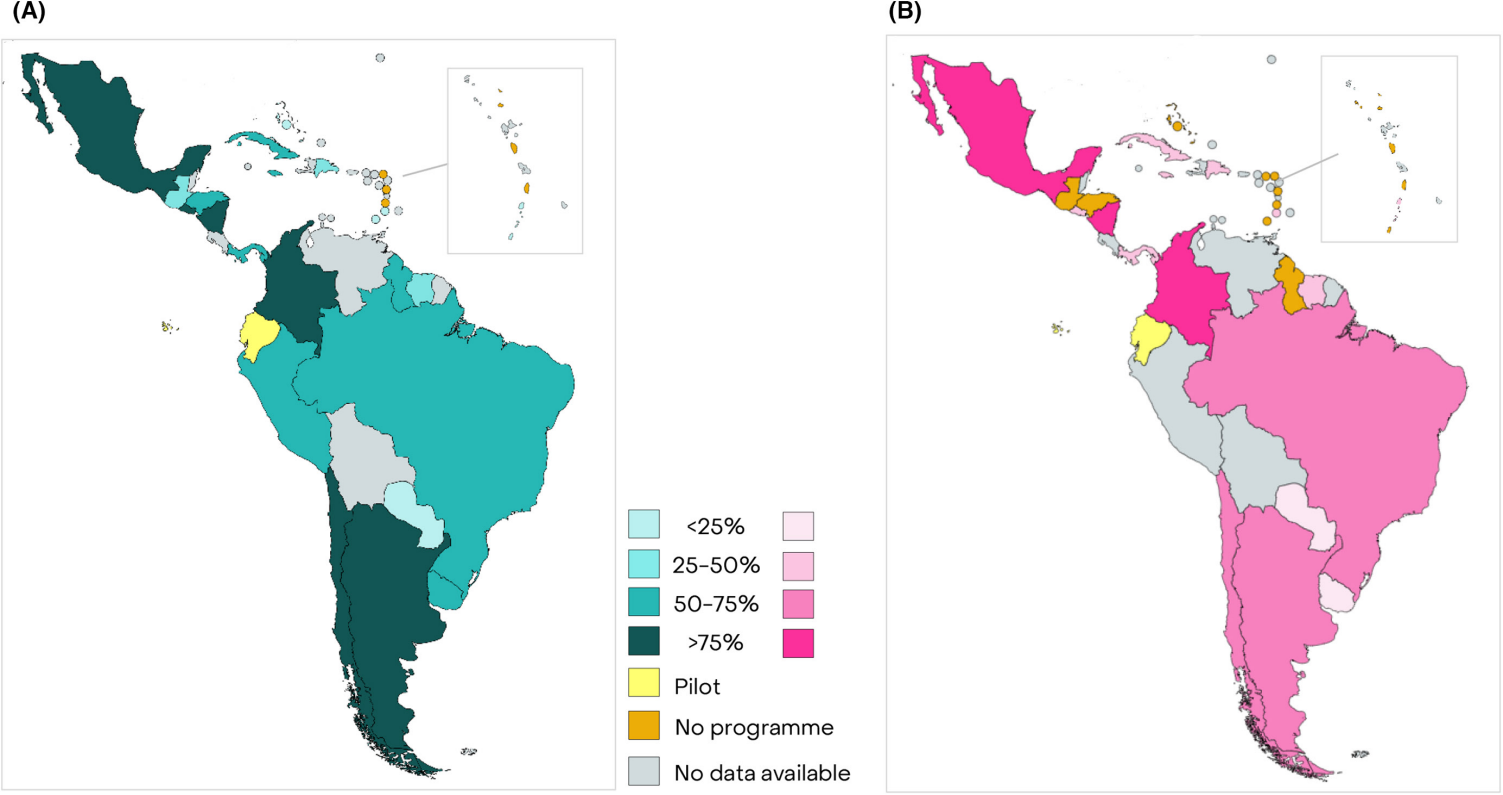
Proportion of essential elements for organized cervical (A) and breast cancer screening (B) programmes that are fulfilled by the programmes in the CELAC.

Argentina, Chile, Colombia, El Salvador, Mexico, and Nicaragua displayed a high level of organization for cervical cancer (>75% of essential elements of organized screening programmes fulfilled), and except for Argentina and El Salvador, they were also highly organized for breast cancer screening (Figure 1A,B). On the other end, cervical cancer screening was poorly organized in Bahamas, Grenada, Paraguay, and SVG; while Paraguay and Uruguay had a low level of organization of their breast cancer screening programmes.

Some countries have a higher level of organization for cervical cancer screening (Argentina, Chile, Colombia, Cuba, Dominican Republic, El Salvador, Jamaica, Nicaragua, Panama, and Uruguay) compared to breast cancer screening. Brazil and SVG were the only countries with a more organized breast cancer screening programme than cervical cancer screening.

For both the cancer sites the essential elements most frequently absent were linkage of screening data with PBCR, system to send individual invitations to eligible population, and publication of performance reports in last 5 years. For breast cancer, another frequent criterion missing was the system allowing the identification of population eligible for screening.

In the countries with a screening programme examined in this paper the UHC service coverage index ranged from 57 (Guatemala) to 80 (Chile, Cuba, and Ecuador), while, when looking at the UHC subindex value on NCD, scores were below 60 in 15 countries (out of 20; 75%). The UHC service coverage showed a significant correlation with the level of organization of cervical cancer screening (*R* ^2^ = 0.3142, *p* = 0.002), but not for breast cancer screening programmes (*R* ^2^ = 0.1382, *p* = 0.237; Figures S1a and S1b).

### 3.5 Availability of quantitative data

**FIGURE 2:**
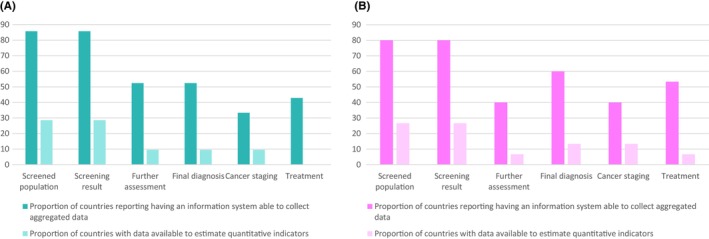
Proportion of countries (%) reporting to have an information system with ability to collect aggregated data and having data available to estimate the quantitative indicators across the screening pathway for cervical (A) and breast cancer (B) in CELAC.

Most of the countries (*N* = 18; 85.7%) having cervical cancer screening programmes reported to be collecting aggregated data on screened population and screening test results, while only 52.4% (*N* = 11) claimed to be collecting data on further assessment and final diagnosis (Figure 2A). No country in the Caribbean referred the collection of aggregated data on cervical cancer staging or treatment of precancers and cancers. When requested, only 6 countries (28.6%) had data available to estimate the number of women screened and the screening test results in the programme. Only two (9.5%) countries could provide aggregate number of women undergoing further assessment, the final diagnosis, and staging of the cancers detected. Available data mainly captured the public sector screening.

As for breast cancer screening, the reported number of programmes collecting data on number screened (*N* = 12; 80.0%), screening test results (*N* = 12; 80.0%), and further assessment (*N* = 6; 40.0%) was lower, but higher for final diagnosis (*N* = 9; 60.0%), staging (*N* = 6; 40.0%) and treatment (*N* = 8; 53.3%; Figure 2B). As observed for cervical cancer screening, actual data available for analysis was quite limited, with only 1 country (6.7%) being able to provide further assessment and treatment data. No Caribbean country had data for analysis of performance of breast cancer screening.

Less than half of the countries reporting to be collecting aggregated data could provide data for analysis. The explanations for such discrepancies put forward by the countries included not recording the information in an effective information system, inability to follow the same women because of a lack of linkage between databases, insufficient human resources to do this linkage, lack of clarity on the target population, incomplete data, etc.

## 4 DISCUSSION

This manuscript reports an in‐depth multi‐country analysis of the policies and organization of breast and cervical cancer screening programmes in CELAC using a common methodology. While most countries conducted cervical cancer screening programmes, such programmes for breast cancer were less frequent in Central America and the Caribbean, and large gaps were noted in the organization of services. Some of the countries in the Caribbean did not have a documented policy and protocol for either screening programme.

A cancer screening programme can reduce cancer incidence (for cervical cancer) and mortality if it has a high coverage and high quality, and can be cost effective and reduce inequity if it is well‐organized and population‐based (with a system of inviting the eligible populations). Invitation has proven to increase participation to screening,^17^, ^18^ but a mechanism to invite the eligible population was rare in the region for either of the screening programmes studied. To maintain high compliance to further assessment, an active follow‐up of screen‐positive women should be in place. The only region where this was present in all countries was Central America for both cancers. Having an active follow‐up of screen‐positive women, as well as a fail‐safe mechanism in place, will increase participation to further assessment.^19^ Moreover, an active follow up can allow health care providers to address reasons for not participating in further assessment, such as fear or fatalism.

WHO recommends breast cancer screening among women 50 to 69 years in well‐resourced settings with strong health systems with the capacity to develop and sustain organized population‐based mammography screening.^20^ In CELAC the only population‐based programme does not rely on mammography, while for those programmes that do, only 5 countries follow this recommendation on target age, and 4 of them on the suggested screening interval of 2 years.

Neither cervical nor breast cancer screening were free of charge in all countries. What raises more concerns is that women had to pay for diagnosis in over 40% of countries and for treatment in over 35% of countries. This results in high economic burden of healthcare in households of those participating in screening, and some women may forgo diagnosis or treatment because of not being able to afford it.^21^

A recent systematic review on breast cancer staging at diagnosis observed considerable heterogeneity among 22 countries from Latin America and the Caribbean in the proportion of patients diagnosed with Stage III‐IV (40.8%, 95% CI 37.0%–44.6%).^22^ Stage at diagnosis is dependent on the efficiency of screening programmes, explains partly the great variation in survival across countries^23^ and influences treatment cost and quality of life. Estimations show that when treating a breast cancer in stage IV the cost can increase 119% compared to stage I.^24^ However, when evaluating the cost of cancer, long term costs of persons surviving the disease should be considered, as well as loss of earnings and loss of productivity for cancer patients and their caregivers. Caring for a cancer patient can impact health,^25^ as well as finances.^26^ Therefore, governments in the CELAC should ensure the universal and timely access of their entire population to screening and downstream services to have a major improvement in their cancer care.^27^, ^28^

Practice of collecting performance data from screening programmes is infrequent in the region, preventing proper monitoring and quality assurance of the programmes. In many countries not having a screening registry collecting individual data hindered being able to track population across the cancer screening pathway, and they had to rely on surveys to provide screening coverage data.^6^ Governments should establish a legal and technical framework together with a functional information system to allow the linkage of databases for health purposes while being aligned with data protection regulations.^29^, ^30^ Countries may interpret differently data protection regulations, but they normally allow this linkage when it is related to the protection of public health,^31^ for which cancer screening and care is a good example. In a region characterized by a fragmentation of health systems^32^ and funding, as well as by a poorly regulated private sector,^33^ linkage of databases can be challenging. Nonetheless, countries need to invest in their information system and have screening data integrated^34^ and linked to a PBCR for adequate cancer control planning.

We observed heterogeneity across Latin American and the Caribbean states, and within regions. For example, cervical and breast cancer screening was free of charge in most countries in Central and South America, while in the Caribbean women had to pay in 33.3% (for cervical cancer) or 50.0% (for breast cancer) of the countries. In both cervical and breast cancer screening, tracking of screen‐positive women was present in all countries in Central America, at least half in the Caribbean and only 33.3% in South America. To better interpret these results, a more in‐depth understanding on the health care delivery, referral systems, proportion of public and private providers, etc. in each country is needed. This will determine whether a country is ready to provide screening services or if it should focus on early diagnosis.

We found there was a significant correlation between the level of organization of cervical cancer screening services and UHC service coverage, but not for breast cancer, which may be explained by the lower number of countries with a breast cancer screening programme. The correlation with cervical cancer screening services shows an important information on how a well‐structured health system impacts the performance of the screening programme. An improvement of the organization of services will require a strengthening of the health system, which can ultimately translate into a higher coverage of essential healthcare services and will result in an increase of cancer screening uptake^35^ and reduction of cancer burden in the countries.

The strengths of this study are the provision of a wide overview of breast and cervical cancer screening organization in CELAC, where not only information is collected directly from the health authorities but also is cross checked against several documents and real practice. Hence, some information may be different in publicly available documents from these countries. A limitation is, that while a high number of countries from CELAC are included in this assessment, 7 out of 33 countries in the region are missing (including large countries such as Bolivia or Venezuela). Additionally, while information came from official sources and went through a rigorous process of validation, we must acknowledge that it may not always reflect reality in the whole country, or policies may still be in the implementation phase. Moreover, the availability of quantitative data for analysis was lower than reported by countries, and it covers mainly the screening activities in the public sector.

## 5 CONCLUSIONS

In this study we saw overall differences by region and by country in both qualitative and quantitative data. Sustainable funding for screening was less of an issue in South America, while access to diagnosis and treatment was more frequently free of charge in Central America. The Caribbean lagged in monitoring and evaluation of the screening programmes. Moreover, the level of organization of cervical cancer screening programmes showed a clear correlation with UHC service coverage.

To improve their screening programmes, countries need pragmatic public health policies, strengthened health systems, sustainable funding, and universal access to cancer diagnosis and treatment. Additionally, countries should enhance their health information system, and ensure adequate monitoring and evaluation. Although recommendations apply to all CELAC, a greater effort is needed in the Caribbean.

## AUTHOR CONTRIBUTIONS

**Isabel Mosquera:** Conceptualization (equal); data curation (equal); formal analysis (lead); investigation (lead); methodology (equal); validation (lead); visualization (lead); writing – original draft (lead). **Clara Barajas:** Investigation (supporting); validation (supporting); writing – review and editing (equal). **Li Zhang:** Formal analysis (supporting); methodology (equal); writing – review and editing (equal). **Eric Lucas:** Data curation (equal); methodology (equal); visualization (supporting); writing – review and editing (equal). **Sara Benitez Majano:** Investigation (supporting); validation (supporting); writing – review and editing (equal). **Mauricio Maza:** Investigation (supporting); writing – review and editing (equal). **Silvana Luciani:** Investigation (supporting); writing – review and editing (equal). **Partha Basu:** Conceptualization (equal); funding acquisition (lead); investigation (lead); methodology (equal); supervision (equal); writing – review and editing (equal). **Andre L Carvalho:** Conceptualization (equal); formal analysis (lead); investigation (lead); methodology (equal); supervision (equal); validation (lead); writing – review and editing (equal).

## FUNDING INFORMATION

This publication is supported by a grant awarded by the Norwegian Research Council (project number 288638) to the Center for Global Health Inequalities Research (CHAIN) at the Norwegian University for Science and Technology (NTNU).

## CONFLICT OF INTEREST STATEMENT

The authors have no conflict of interest to declare.

## ETHICS STATEMENT

Not applicable.

## DISCLAIMER

Where authors are identified as personnel of the International Agency for Research on Cancer/World Health Organization, the authors alone are responsible for the views expressed in this article and they do not necessarily represent the decisions, policy, or views of the International Agency for Research on Cancer/World Health Organization.

## Supporting information


Appendix S1:

## Data Availability

The datasets generated and/or analyzed during the current study are included within the article, and mostly accessible at canscreen5.iarc.fr.

## References

[1] Ferlay J , Ervik M , Lam F , et al. Global Cancer Observatory: Cancer Today. 2022 Accessed November 12, 2022. https://gco.iarc.fr/today

[2] Jansen EEL , Zielonke N , Gini A , et al. Effect of organised cervical cancer screening on cervical cancer mortality in Europe: a systematic review. Eur J Cancer. 2020;127:207‐223. 31980322 10.1016/j.ejca.2019.12.013

[3] Zielonke N , Gini A , Jansen EEL , et al. Evidence for reducing cancer‐specific mortality due to screening for breast cancer in Europe: A systematic review. Eur J Cancer. 2020;127:191‐206. 31932175 10.1016/j.ejca.2019.12.010

[4] IARC . Breast cancer screening, IARC Handbook on Cancer Prevention. 15 2016.

[5] Murillo R , Herrero R , Sierra MS , Forman D . Cervical cancer in Central and South America: Burden of disease and status of disease control. Cancer Epidemiol. 2016;44(Suppl 1):S121‐S130. 27678314 10.1016/j.canep.2016.07.015

[6] Bruni L , Serrano B , Roura E , et al. Cervical cancer screening programmes and age‐specific coverage estimates for 202 countries and territories worldwide: a review and synthetic analysis. Lancet Glob Health. 2022;10(8):e1115‐e1127. 35839811 10.1016/S2214-109X(22)00241-8 PMC9296658

[7] IARC . Cervical cancer screening, IARC Handbook on Cancer Prevention. 18 2022.

[8] Secretaria de Salud México . Caminando a la excelencia. Cierre; 2019.

[9] Zhang L , Carvalho AL , Mosquera I , et al. An international consensus on the essential and desirable criteria for an ‘organized’ cancer screening programme. BMC Med. 2022;20(1):101. 35317783 10.1186/s12916-022-02291-7 PMC8941752

[10] IARC . Cancer Screening in Five Continents. Accessed November 21, 2022. https://canscreen5.iarc.fr

[11] Lucas E , Carvalho AL , Basu P . Cancer Screening in Five Continents (CanScreen5): a project designed to improve the quality of cancer screening programmes. Cancer Control 2019 Cancer care in emerging health systems. 2019;6:44‐48.

[12] Zhang L , Mosquera I , Lucas E , Rol ML , Carvalho AL , Basu P . CanScreen5, a global repository for breast, cervical and colorectal cancer screening programs. Nat Med. 2023;29(5):1135‐1145. 37106168 10.1038/s41591-023-02315-6 PMC10202799

[13] IARC Learning . Improving the quality of cancer screening. Self‐paced learning programme . Accessed November 12, 2022. https://learning.iarc.fr/edp/courses/pgm‐cancer‐screening/

[14] Harris PA , Taylor R , Thielke R , Payne J , Gonzalez N , Conde JG . Research electronic data capture (REDCap)–a metadata‐driven methodology and workflow process for providing translational research informatics support. J Biomed Inform. 2009;42(2):377‐381. 18929686 10.1016/j.jbi.2008.08.010 PMC2700030

[15] Harris PA , Taylor R , Minor BL , et al. The REDCap consortium: Building an international community of software platform partners. J Biomed Inform. 2019;95:103208. 31078660 10.1016/j.jbi.2019.103208 PMC7254481

[16] WHO. The Global Health Observatory: UHC Service Coverage Index (SDG 3.8.1). Accessed November 12, 2022. https://www.who.int/data/gho/data/indicators/indicator‐details/GHO/uhc‐index‐of‐service‐coverage

[17] Quinn M , Babb P , Jones J , Allen E . Effect of screening on incidence of and mortality from cancer of cervix in England: evaluation based on routinely collected statistics. Bmj. 1999;318(7188):904‐908. 10102852 10.1136/bmj.318.7188.904 PMC27810

[18] Mandrik O , Tolma E , Zielonke N , et al. Systematic reviews as a “lens of evidence”: Determinants of participation in breast cancer screening. J Med Screen. 2021;28(2):70‐79. 32517538 10.1177/0969141320930743 PMC8167916

[19] Miles A , Cockburn J , Smith RA , Wardle J . A perspective from countries using organized screening programs. Cancer. 2004;101(5 Suppl):1201‐1213. 15316915 10.1002/cncr.20505

[20] WHO. WHO position paper on mammography screening . 2014. Accessed June 26, 2023. https://www.who.int/publications/i/item/who‐position‐paper‐on‐mammography‐screening 25642524

[21] Murphy A , Palafox B , Walli‐Attaei M , et al. The household economic burden of non‐communicable diseases in 18 countries. BMJ Glob Health. 2020;5(2):e002040. 10.1136/bmjgh-2019-002040 PMC7042605 32133191

[22] de Lemos LLP , Carvalho de Souza M , Pena Moreira D , et al. Stage at diagnosis and stage‐specific survival of breast cancer in Latin America and the Caribbean: A systematic review and meta‐analysis. PLoS One. 2019;14(10):e0224012. 31618268 10.1371/journal.pone.0224012 PMC6799865

[23] Walters S , Maringe C , Butler J , et al. Breast cancer survival and stage at diagnosis in Australia, Canada, Denmark, Norway, Sweden and the UK, 2000–2007: a population‐based study. Br J Cancer. 2013;108(5):1195‐1208. 23449362 10.1038/bjc.2013.6 PMC3619080

[24] Palacios A , Rojas‐Roque C , González L , et al. Direct Medical Costs, Productivity Loss Costs and Out‐Of‐Pocket Expenditures in Women with Breast Cancer in Latin America and the Caribbean: A Systematic Review. Pharmacoeconomics. 2021;39(5):485‐502. 33782865 10.1007/s40273-021-01014-9

[25] Pinquart M , Sörensen S . Differences between caregivers and noncaregivers in psychological health and physical health: a meta‐analysis. Psychol Aging. 2003;18(2):250‐267. 12825775 10.1037/0882-7974.18.2.250

[26] Gardiner C , Brereton L , Frey R , Wilkinson‐Meyers L , Gott M . Exploring the financial impact of caring for family members receiving palliative and end‐of‐life care: a systematic review of the literature. Palliat Med. 2014;28(5):375‐390. 24201134 10.1177/0269216313510588

[27] Organización Panamericana de la Salud . Plan de acción sobre la prevención y el control del cáncer cervicouterino 2018–2030. OPS; 2018.

[28] American Cancer Society . Universal health coverage. The Cancer Atlas. edn; 2019.

[29] Anttila A , Lönnberg S , Ponti A , et al. Towards better implementation of cancer screening in Europe through improved monitoring and evaluation and greater engagement of cancer registries. Eur J Cancer. 2015;51(2):241‐251. 25483785 10.1016/j.ejca.2014.10.022

[30] OEA. Departamento de Derecho Internacional. Protección de Datos Personales . Accessed November 12, 2022. https://www.oas.org/es/sla/ddi/proteccion_datos_personales.asp

[31] OEA. Principios actualizados sobre la privacidad y la protección de datos personales . 2021. Accessed June 26, 2023. https://www.oas.org/es/sla/cji/docs/Publicacion_Proteccion_Datos_Personales_Principios_Actualizados_2021.pdf

[32] Frenk J , Gómez‐Dantés O . Health Systems in Latin America: The Search for Universal Health Coverage. Arch Med Res. 2018;49(2):79‐83. 29960828 10.1016/j.arcmed.2018.06.002

[33] Atun R , de Andrade LO , Almeida G , et al. Health‐system reform and universal health coverage in Latin America. Lancet. 2015;385(9974):1230‐1247. 25458725 10.1016/S0140-6736(14)61646-9

[34] Vale DB , Teixeira JC , Bragança JF , Derchain S , Sarian LO , Zeferino LC . Elimination of cervical cancer in low‐ and middle‐income countries: Inequality of access and fragile healthcare systems. Int J Gynaecol Obstet. 2021;152(1):7‐11. 33128771 10.1002/ijgo.13458

[35] Hatch B , Hoopes M , Darney BG , et al. Impacts of the Affordable Care Act on Receipt of Women's Preventive Services in Community Health Centers in Medicaid Expansion and Nonexpansion States. Womens Health Issues. 2021;31(1):9‐16. 33023807 10.1016/j.whi.2020.08.011 PMC9206529

