# Correction to “Assessment of organization of cervical and breast cancer screening programmes in the Latin American and the Caribbean states: The CanScreen5 framework”

**DOI:** 10.1002/cam4.6775

**Published:** 2023-12-19

Mosquera I, Barajas CB, Zhang L, et al. Assessment of organization of cervical and breast cancer screening programmes in the Latin American and the Caribbean states: The CanScreen5 framework. Cancer Med. 2023;12:19935‐19948. doi:10.1002/cam4.6492

In the originally published article, in Table 3, the screening interval has been presented as ‘3 years’ for Chile (2020) under HPV & cytology (co‐test). This has been corrected to ‘5 years.’

We apologize for this error.

