# Supplementary material for: Assessment of organization of cervical and breast cancer screening programmes in the Latin American and the Caribbean states: The CanScreen5 framework
Source: Cancer Med. 2023 Sep 28;12(19):19935–48. doi: 10.1002/cam4.6492 (PMC10587918; doi:10.1002/cam4.6492)
Supplement: Supplementary file 1 — Appendix S1: [file CAM4-12-19935-s001.pdf]

Supplementary figure 1. Dispersion graphs of coverage of essential health services and level of organization of cervical cancer (1.a) and breast cancer screening programmes (1.b) by region in CELAC.

1.a

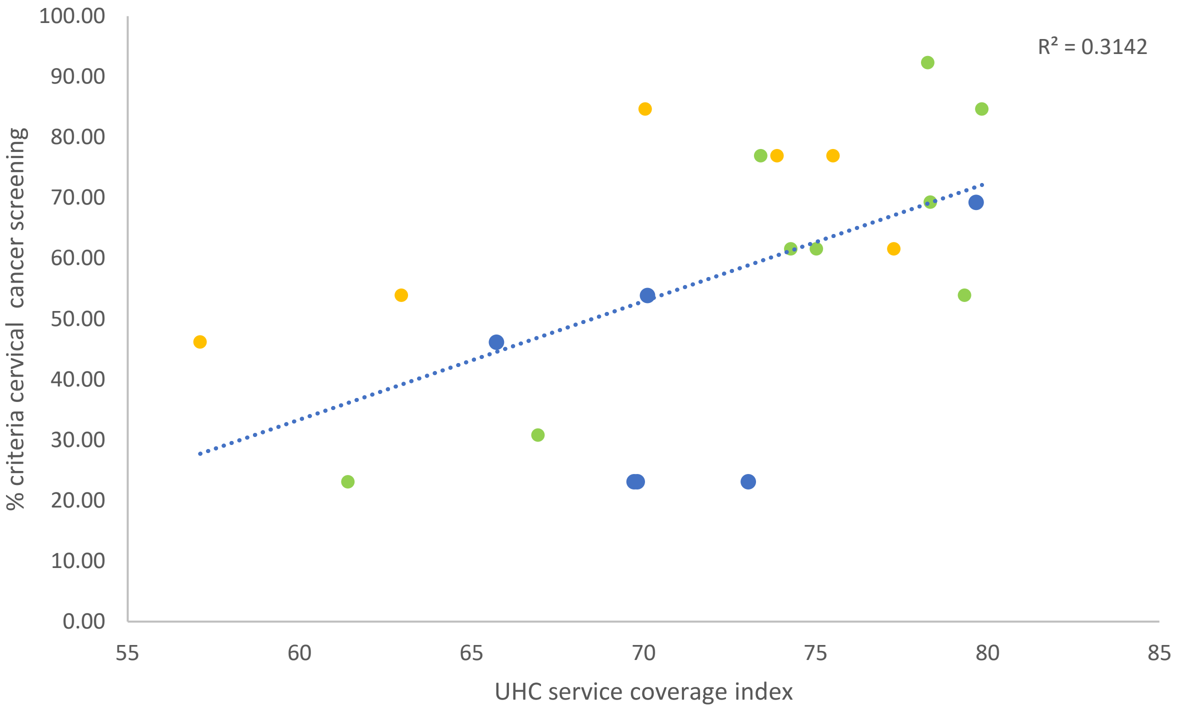

p=0.002

1.b

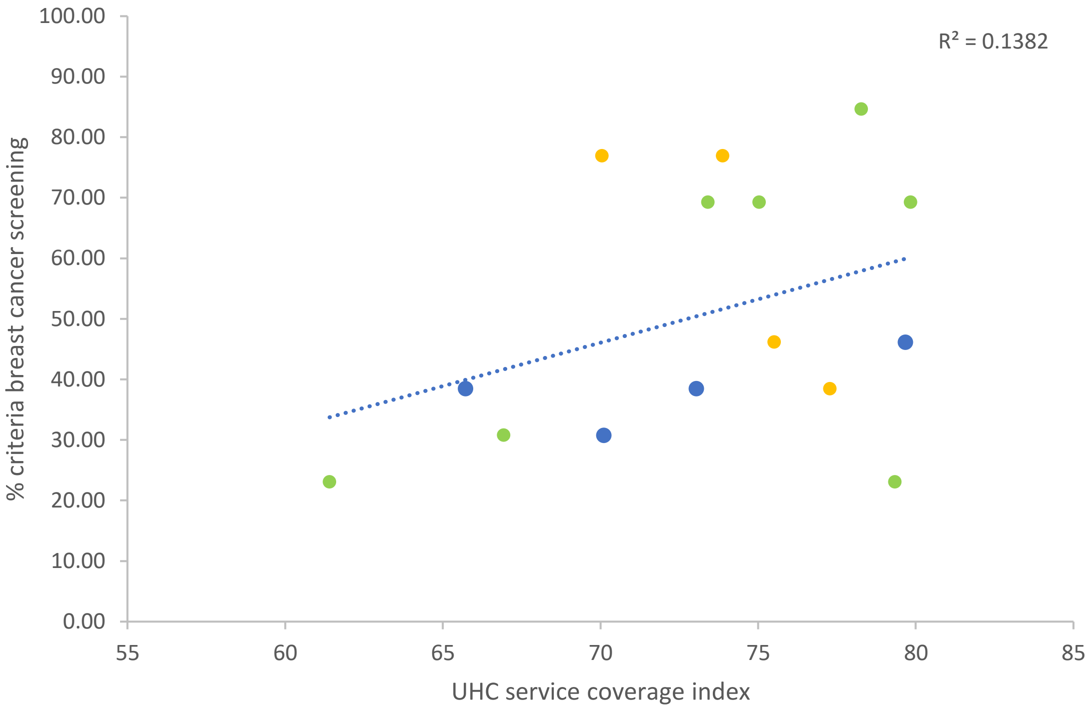

p=0.237

Central America; South America; The Caribbean
